# Supplementary material for: Decreased brain connectivity in smoking contrasts with increased connectivity in drinking
Source: eLife. 2019 Jan 8;8:e40765. doi: 10.7554/eLife.40765 (PMC6336408; doi:10.7554/eLife.40765)
Supplement: Figure 2—source data 1. [file elife-40765-fig2-data1.docx]

**Figure 2—figure source data 1.** All significant links (FDR p<0.05) between the AAL2 areas for the smoking group.

| **Functional connectivity** | | **t value** | **p value** | **Functional connectivity** | | **t value** | **p value** |
| --- | --- | --- | --- | --- | --- | --- | --- |
| Frontal_Sup_2_L | Frontal_Mid_2_R | -4.409 | 1.23E-05 | Frontal_Inf_Oper_L | Precuneus_L | -3.475 | 5.48E-04 |
| Frontal_Sup_2_R | Frontal_Mid_2_R | -3.912 | 1.02E-04 | SupraMarginal_R | Precuneus_L | -3.472 | 5.55E-04 |
| Frontal_Sup_2_L | Frontal_Inf_Oper_R | -4.354 | 1.57E-05 | Frontal_Inf_Orb_2_L | Supp_Motor_Area_R | -3.452 | 5.97E-04 |
| Frontal_Sup_2_R | Frontal_Inf_Oper_R | -3.980 | 7.75E-05 | Rolandic_Oper_L | Frontal_Med_Orb_R | -3.453 | 5.93E-04 |
| Frontal_Mid_2_L | Frontal_Inf_Oper_R | -3.970 | 8.07E-05 | Parietal_Sup_L | Parietal_Sup_R | -3.461 | 5.77E-04 |
| Frontal_Inf_Oper_R | Frontal_Inf_Tri_L | -4.271 | 2.26E-05 | Rolandic_Oper_L | Precuneus_R | -3.458 | 5.84E-04 |
| Frontal_Inf_Orb_2_L | Rolandic_Oper_L | -3.933 | 9.37E-05 | Rolandic_Oper_L | Heschl_R | -3.457 | 5.85E-04 |
| Frontal_Inf_Orb_2_L | Rolandic_Oper_R | -4.019 | 6.60E-05 | ParaHippocampal_L | Temporal_Sup_L | -3.454 | 5.91E-04 |
| Frontal_Sup_2_L | OFCmed_R | -4.464 | 9.63E-06 | Frontal_Med_Orb_R | Fusiform_L | -3.445 | 6.11E-04 |
| Frontal_Inf_Oper_L | OFClat_R | -3.935 | 9.31E-05 | Insula_R | Amygdala_R | -3.441 | 6.21E-04 |
| Frontal_Inf_Orb_2_L | Insula_R | -3.973 | 7.95E-05 | Putamen_L | Pallidum_L | -3.438 | 6.27E-04 |
| Frontal_Inf_Oper_L | Cingulate_Ant_R | -3.914 | 1.01E-04 | OFCpost_L | SupraMarginal_R | -3.432 | 6.41E-04 |
| Frontal_Inf_Orb_2_L | Amygdala_R | -4.067 | 5.41E-05 | Frontal_Inf_Orb_2_R | Insula_L | -3.429 | 6.49E-04 |
| Frontal_Inf_Orb_2_L | Occipital_Mid_R | -3.984 | 7.60E-05 | Frontal_Inf_Tri_L | Precuneus_L | -3.425 | 6.57E-04 |
| Frontal_Inf_Orb_2_L | Fusiform_R | -3.865 | 1.23E-04 | OFClat_R | Thalamus_L | -3.427 | 6.53E-04 |
| Frontal_Inf_Orb_2_L | Parietal_Sup_L | -3.861 | 1.25E-04 | Frontal_Mid_2_R | Frontal_Inf_Orb_2_L | -3.423 | 6.62E-04 |
| Frontal_Inf_Orb_2_R | Parietal_Sup_L | -3.955 | 8.59E-05 | Frontal_Inf_Oper_R | Cingulate_Post_R | -3.409 | 6.97E-04 |
| Frontal_Sup_2_L | Parietal_Sup_R | -4.001 | 7.11E-05 | Frontal_Med_Orb_R | Occipital_Inf_L | -3.404 | 7.08E-04 |
| Frontal_Inf_Orb_2_L | Parietal_Sup_R | -4.152 | 3.78E-05 | Amygdala_R | Fusiform_L | -3.416 | 6.78E-04 |
| Frontal_Med_Orb_R | Parietal_Sup_R | -4.147 | 3.86E-05 | Frontal_Inf_Tri_L | Fusiform_R | -3.402 | 7.15E-04 |
| Rectus_L | Parietal_Sup_R | -3.870 | 1.21E-04 | Frontal_Inf_Tri_R | Parietal_Sup_L | -3.414 | 6.83E-04 |
| OFCmed_R | Parietal_Sup_R | -4.080 | 5.11E-05 | Cingulate_Post_L | Parietal_Sup_R | -3.401 | 7.16E-04 |
| Frontal_Inf_Tri_L | Parietal_Inf_L | -3.878 | 1.17E-04 | Amygdala_R | Parietal_Inf_L | -3.412 | 6.89E-04 |
| Frontal_Inf_Tri_R | Parietal_Inf_L | -3.861 | 1.25E-04 | OFCmed_R | SupraMarginal_L | -3.400 | 7.19E-04 |
| Frontal_Inf_Tri_L | Parietal_Inf_R | -3.885 | 1.14E-04 | OFCant_R | Precuneus_L | -3.397 | 7.26E-04 |
| Frontal_Inf_Tri_L | SupraMarginal_R | -4.090 | 4.90E-05 | Frontal_Sup_2_L | Putamen_R | -3.416 | 6.79E-04 |
| Frontal_Inf_Orb_2_L | SupraMarginal_R | -4.297 | 2.02E-05 | Occipital_Mid_L | Temporal_Sup_L | -3.417 | 6.77E-04 |
| Parietal_Sup_R | Precuneus_L | -4.014 | 6.74E-05 | Occipital_Mid_R | Temporal_Sup_L | -3.397 | 7.25E-04 |
| Frontal_Inf_Tri_L | Precuneus_R | -3.929 | 9.54E-05 | Fusiform_R | Temporal_Sup_L | -3.397 | 7.27E-04 |
| Caudate_L | Putamen_L | -4.216 | 2.87E-05 | Fusiform_R | Temporal_Pole_Sup_L | -3.403 | 7.10E-04 |
| Caudate_R | Putamen_R | -3.972 | 7.99E-05 | Rolandic_Oper_R | Amygdala_R | -3.394 | 7.34E-04 |
| Caudate_L | Pallidum_L | -4.255 | 2.42E-05 | Frontal_Inf_Orb_2_L | Calcarine_L | -3.387 | 7.53E-04 |
| Frontal_Inf_Orb_2_L | Temporal_Sup_L | -4.106 | 4.59E-05 | Frontal_Med_Orb_L | Occipital_Sup_R | -3.386 | 7.55E-04 |
| Hippocampus_L | Temporal_Sup_L | -4.179 | 3.37E-05 | Hippocampus_R | Occipital_Mid_R | -3.391 | 7.41E-04 |
| Frontal_Inf_Orb_2_L | Temporal_Sup_R | -4.317 | 1.85E-05 | Frontal_Inf_Oper_R | Precuneus_R | -3.387 | 7.53E-04 |
| Hippocampus_L | Temporal_Sup_R | -3.863 | 1.24E-04 | Rolandic_Oper_R | Heschl_R | -3.387 | 7.54E-04 |
| Hippocampus_R | Temporal_Sup_R | -3.909 | 1.03E-04 | Precuneus_R | Temporal_Sup_L | -3.385 | 7.58E-04 |
| Amygdala_R | Temporal_Sup_R | -3.945 | 8.94E-05 | Frontal_Mid_2_R | Cingulate_Ant_R | -3.382 | 7.66E-04 |
| Frontal_Sup_2_L | OFCant_R | -3.831 | 1.41E-04 | Hippocampus_R | Parietal_Sup_R | -3.382 | 7.68E-04 |
| Rolandic_Oper_L | Hippocampus_L | -3.833 | 1.40E-04 | Frontal_Inf_Tri_L | Cingulate_Mid_R | -3.375 | 7.85E-04 |
| Rectus_R | Parietal_Sup_R | -3.834 | 1.40E-04 | Frontal_Inf_Orb_2_L | Calcarine_R | -3.376 | 7.83E-04 |
| Frontal_Inf_Oper_R | Parietal_Inf_L | -3.833 | 1.40E-04 | Frontal_Inf_Orb_2_R | Temporal_Sup_R | -3.377 | 7.80E-04 |
| Frontal_Sup_2_L | Frontal_Inf_Oper_L | -3.751 | 1.93E-04 | Frontal_Inf_Tri_L | Insula_R | -3.372 | 7.95E-04 |
| Frontal_Inf_Oper_R | Frontal_Inf_Orb_2_L | -3.770 | 1.79E-04 | OFClat_L | SupraMarginal_R | -3.373 | 7.92E-04 |
| Frontal_Inf_Orb_2_L | Insula_L | -3.805 | 1.56E-04 | Frontal_Mid_2_L | Frontal_Inf_Orb_2_R | -3.368 | 8.06E-04 |
| Frontal_Inf_Oper_L | Amygdala_R | -3.737 | 2.04E-04 | Supp_Motor_Area_R | OFCmed_R | -3.348 | 8.64E-04 |
| Frontal_Inf_Tri_L | Amygdala_R | -3.800 | 1.60E-04 | Frontal_Mid_2_R | Cingulate_Post_L | -3.347 | 8.69E-04 |
| Frontal_Inf_Orb_2_L | Lingual_L | -3.746 | 1.97E-04 | Frontal_Inf_Oper_L | Cingulate_Post_R | -3.350 | 8.58E-04 |
| Frontal_Inf_Orb_2_L | Occipital_Mid_L | -3.782 | 1.71E-04 | Frontal_Inf_Orb_2_L | ParaHippocampal_R | -3.357 | 8.39E-04 |
| Frontal_Inf_Tri_L | Parietal_Sup_R | -3.733 | 2.07E-04 | Cingulate_Mid_L | Amygdala_R | -3.354 | 8.48E-04 |
| Frontal_Inf_Orb_2_R | Parietal_Inf_L | -3.734 | 2.06E-04 | OFCmed_R | Occipital_Sup_L | -3.354 | 8.48E-04 |
| Frontal_Mid_2_R | Precuneus_L | -3.777 | 1.75E-04 | Frontal_Inf_Orb_2_L | Occipital_Inf_L | -3.347 | 8.69E-04 |
| OFCmed_R | Precuneus_L | -3.737 | 2.04E-04 | Parietal_Sup_R | Angular_L | -3.357 | 8.37E-04 |
| Frontal_Inf_Orb_2_L | Precuneus_R | -3.735 | 2.06E-04 | Putamen_L | Pallidum_R | -3.348 | 8.67E-04 |
| Putamen_L | Putamen_R | -3.743 | 2.00E-04 | OFCpost_L | Temporal_Sup_R | -3.355 | 8.43E-04 |
| Hippocampus_L | Heschl_L | -3.808 | 1.55E-04 | Frontal_Inf_Oper_L | Temporal_Inf_R | -3.356 | 8.42E-04 |
| Frontal_Inf_Orb_2_L | Heschl_R | -3.739 | 2.03E-04 | OFClat_L | Amygdala_R | -3.342 | 8.83E-04 |
| Hippocampus_R | Temporal_Sup_L | -3.806 | 1.56E-04 | Frontal_Inf_Oper_R | Cingulate_Ant_R | -3.332 | 9.14E-04 |
| Amygdala_R | Temporal_Sup_L | -3.749 | 1.95E-04 | Supp_Motor_Area_R | Hippocampus_L | -3.333 | 9.14E-04 |
| Fusiform_L | Temporal_Sup_L | -3.792 | 1.65E-04 | Hippocampus_R | Occipital_Inf_L | -3.332 | 9.15E-04 |
| Hippocampus_R | Temporal_Pole_Sup_L | -3.743 | 2.00E-04 | Precuneus_L | Temporal_Sup_L | -3.328 | 9.29E-04 |
| Frontal_Inf_Oper_L | Frontal_Inf_Orb_2_R | -3.724 | 2.14E-04 | Rectus_R | Occipital_Sup_R | -3.324 | 9.42E-04 |
| Frontal_Inf_Oper_L | OFCpost_R | -3.701 | 2.34E-04 | OFCmed_R | Occipital_Mid_R | -3.326 | 9.36E-04 |
| Frontal_Inf_Orb_2_L | Cingulate_Mid_L | -3.701 | 2.35E-04 | Lingual_L | Paracentral_Lobule_R | -3.323 | 9.44E-04 |
| Supp_Motor_Area_R | Amygdala_R | -3.707 | 2.29E-04 | Frontal_Inf_Oper_L | Hippocampus_R | -3.321 | 9.53E-04 |
| Frontal_Inf_Orb_2_L | Fusiform_L | -3.706 | 2.30E-04 | Frontal_Mid_2_R | Frontal_Inf_Tri_L | -3.293 | 1.05E-03 |
| Frontal_Inf_Oper_R | Precuneus_L | -3.714 | 2.23E-04 | Frontal_Mid_2_L | Frontal_Inf_Tri_R | -3.295 | 1.04E-03 |
| Frontal_Inf_Oper_R | Frontal_Inf_Tri_R | -3.694 | 2.41E-04 | Frontal_Inf_Oper_R | Frontal_Med_Orb_R | -3.311 | 9.85E-04 |
| Frontal_Mid_2_L | Frontal_Mid_2_R | -3.679 | 2.55E-04 | Frontal_Sup_2_L | Insula_R | -3.292 | 1.05E-03 |
| Frontal_Inf_Orb_2_L | Hippocampus_R | -3.674 | 2.60E-04 | Frontal_Inf_Tri_L | Cingulate_Mid_L | -3.311 | 9.87E-04 |
| Frontal_Inf_Orb_2_L | Occipital_Sup_L | -3.673 | 2.61E-04 | Rolandic_Oper_L | Cingulate_Post_R | -3.298 | 1.03E-03 |
| Rolandic_Oper_L | Precuneus_L | -3.670 | 2.65E-04 | Rolandic_Oper_L | Hippocampus_R | -3.301 | 1.02E-03 |
| Hippocampus_R | Fusiform_R | -3.665 | 2.69E-04 | Insula_L | Amygdala_R | -3.301 | 1.02E-03 |
| OFCmed_R | Parietal_Sup_L | -3.661 | 2.74E-04 | Frontal_Inf_Tri_L | Calcarine_L | -3.308 | 9.96E-04 |
| OFCpost_L | Heschl_L | -3.659 | 2.76E-04 | OFCmed_R | Occipital_Sup_R | -3.290 | 1.06E-03 |
| Frontal_Med_Orb_R | Occipital_Sup_R | -3.655 | 2.80E-04 | Frontal_Mid_2_L | Parietal_Sup_R | -3.290 | 1.06E-03 |
| Frontal_Sup_2_L | SupraMarginal_R | -3.650 | 2.85E-04 | Cingulate_Ant_R | Parietal_Inf_L | -3.306 | 1.00E-03 |
| OFClat_L | Parietal_Sup_R | -3.633 | 3.04E-04 | Olfactory_R | SupraMarginal_L | -3.293 | 1.05E-03 |
| Caudate_L | Putamen_R | -3.634 | 3.03E-04 | Frontal_Inf_Orb_2_R | SupraMarginal_R | -3.307 | 1.00E-03 |
| Frontal_Med_Orb_R | Temporal_Sup_R | -3.640 | 2.96E-04 | Fusiform_L | Temporal_Sup_R | -3.302 | 1.02E-03 |
| Frontal_Sup_2_R | Frontal_Inf_Oper_L | -3.609 | 3.33E-04 | Hippocampus_L | Temporal_Pole_Sup_L | -3.295 | 1.04E-03 |
| Frontal_Sup_2_L | Frontal_Inf_Orb_2_R | -3.604 | 3.40E-04 | Frontal_Inf_Orb_2_L | Temporal_Pole_Sup_R | -3.313 | 9.79E-04 |
| Supp_Motor_Area_L | OFCmed_R | -3.612 | 3.29E-04 | Hippocampus_R | Temporal_Pole_Sup_R | -3.298 | 1.03E-03 |
| Rolandic_Oper_R | Hippocampus_L | -3.605 | 3.38E-04 | Hippocampus_R | Temporal_Mid_R | -3.304 | 1.01E-03 |
| Hippocampus_R | Occipital_Sup_R | -3.613 | 3.29E-04 | Hippocampus_R | Occipital_Inf_R | -3.286 | 1.07E-03 |
| OFCant_R | Parietal_Sup_L | -3.599 | 3.46E-04 | Frontal_Inf_Tri_L | Fusiform_L | -3.284 | 1.08E-03 |
| Frontal_Sup_2_L | Parietal_Inf_R | -3.597 | 3.48E-04 | Supp_Motor_Area_L | Precuneus_R | -3.283 | 1.09E-03 |
| Frontal_Inf_Oper_L | Precuneus_R | -3.622 | 3.18E-04 | Frontal_Inf_Tri_L | Frontal_Inf_Tri_R | -3.261 | 1.17E-03 |
| Hippocampus_R | Paracentral_Lobule_R | -3.602 | 3.42E-04 | Frontal_Inf_Orb_2_L | OFCmed_R | -3.271 | 1.13E-03 |
| Frontal_Inf_Orb_2_L | Heschl_L | -3.598 | 3.47E-04 | Rolandic_Oper_L | OFCpost_L | -3.272 | 1.13E-03 |
| Frontal_Sup_Medial_R | Temporal_Sup_R | -3.607 | 3.35E-04 | Frontal_Sup_2_L | OFClat_R | -3.250 | 1.22E-03 |
| Frontal_Inf_Oper_R | OFClat_L | -3.583 | 3.67E-04 | OFClat_R | Insula_L | -3.267 | 1.15E-03 |
| Frontal_Inf_Orb_2_R | Parietal_Sup_R | -3.583 | 3.68E-04 | Frontal_Mid_2_L | Cingulate_Ant_R | -3.256 | 1.20E-03 |
| Lingual_L | Temporal_Sup_L | -3.579 | 3.73E-04 | Supp_Motor_Area_R | Hippocampus_R | -3.254 | 1.20E-03 |
| Cuneus_R | Paracentral_Lobule_R | -3.573 | 3.82E-04 | Rolandic_Oper_L | ParaHippocampal_L | -3.254 | 1.20E-03 |
| Hippocampus_R | Lingual_L | -3.566 | 3.92E-04 | Frontal_Inf_Orb_2_R | Amygdala_R | -3.262 | 1.17E-03 |
| Frontal_Inf_Tri_L | Parietal_Sup_L | -3.566 | 3.92E-04 | Frontal_Inf_Tri_L | Lingual_L | -3.273 | 1.13E-03 |
| Pallidum_L | Pallidum_R | -3.564 | 3.95E-04 | Rectus_R | Occipital_Sup_L | -3.260 | 1.18E-03 |
| Frontal_Sup_2_L | Frontal_Inf_Tri_R | -3.534 | 4.41E-04 | OFClat_L | Occipital_Mid_R | -3.265 | 1.16E-03 |
| Precentral_R | Frontal_Inf_Orb_2_L | -3.502 | 4.96E-04 | Frontal_Inf_Tri_L | Occipital_Inf_L | -3.260 | 1.18E-03 |
| Frontal_Inf_Oper_R | Frontal_Inf_Orb_2_R | -3.507 | 4.88E-04 | Frontal_Sup_Medial_R | Parietal_Sup_R | -3.244 | 1.24E-03 |
| Frontal_Inf_Oper_L | Frontal_Med_Orb_R | -3.501 | 4.98E-04 | OFCant_R | Parietal_Sup_R | -3.269 | 1.14E-03 |
| Frontal_Inf_Orb_2_L | Cingulate_Mid_R | -3.504 | 4.93E-04 | OFCpost_L | Parietal_Sup_R | -3.275 | 1.12E-03 |
| Frontal_Inf_Tri_R | Amygdala_R | -3.498 | 5.03E-04 | OFCpost_R | Parietal_Sup_R | -3.249 | 1.22E-03 |
| Rolandic_Oper_L | Lingual_L | -3.509 | 4.83E-04 | OFCpost_R | Parietal_Inf_L | -3.276 | 1.11E-03 |
| Hippocampus_L | Lingual_L | -3.523 | 4.59E-04 | Frontal_Inf_Tri_R | Precuneus_L | -3.246 | 1.24E-03 |
| Frontal_Inf_Orb_2_L | Lingual_R | -3.553 | 4.11E-04 | Rolandic_Oper_R | Precuneus_L | -3.252 | 1.21E-03 |
| Hippocampus_R | Occipital_Sup_L | -3.499 | 5.01E-04 | Supp_Motor_Area_R | Precuneus_R | -3.247 | 1.23E-03 |
| Frontal_Inf_Orb_2_L | Occipital_Sup_R | -3.504 | 4.93E-04 | Cingulate_Mid_L | Precuneus_R | -3.265 | 1.16E-03 |
| Rectus_L | Occipital_Sup_R | -3.497 | 5.06E-04 | SupraMarginal_L | Precuneus_R | -3.248 | 1.23E-03 |
| Frontal_Inf_Tri_L | Occipital_Mid_L | -3.548 | 4.19E-04 | Cuneus_L | Paracentral_Lobule_R | -3.249 | 1.22E-03 |
| Frontal_Inf_Tri_L | Occipital_Mid_R | -3.542 | 4.28E-04 | OFCpost_L | Heschl_R | -3.270 | 1.14E-03 |
| Hippocampus_R | Fusiform_L | -3.531 | 4.46E-04 | Hippocampus_L | Temporal_Pole_Sup_R | -3.260 | 1.18E-03 |
| Amygdala_R | Parietal_Sup_R | -3.530 | 4.48E-04 | Occipital_Mid_R | Temporal_Pole_Sup_R | -3.244 | 1.24E-03 |
| OFCant_R | Parietal_Inf_L | -3.516 | 4.71E-04 | Fusiform_L | Temporal_Pole_Sup_R | -3.244 | 1.24E-03 |
| Frontal_Mid_2_L | SupraMarginal_R | -3.552 | 4.12E-04 | Hippocampus_R | Temporal_Mid_L | -3.261 | 1.17E-03 |
| Hippocampus_L | SupraMarginal_R | -3.536 | 4.37E-04 | Temporal_Sup_L | Temporal_Pole_Mid_L | -3.244 | 1.24E-03 |
| Amygdala_R | SupraMarginal_R | -3.519 | 4.66E-04 | SupraMarginal_R | Temporal_Inf_L | -3.245 | 1.24E-03 |
| SupraMarginal_L | Precuneus_L | -3.502 | 4.97E-04 | OFCpost_L | Insula_R | -3.241 | 1.26E-03 |
| OFCmed_R | Precuneus_R | -3.495 | 5.09E-04 | Rolandic_Oper_L | Lingual_R | -3.241 | 1.26E-03 |
| Frontal_Mid_2_L | Putamen_R | -3.509 | 4.84E-04 | Hippocampus_R | Heschl_L | -3.237 | 1.28E-03 |
| Frontal_Med_Orb_R | Temporal_Sup_L | -3.511 | 4.81E-04 | Precuneus_L | Heschl_L | -3.236 | 1.28E-03 |
| OFCpost_L | Temporal_Sup_L | -3.535 | 4.40E-04 | ParaHippocampal_R | Temporal_Sup_L | -3.238 | 1.27E-03 |
| Occipital_Mid_R | Temporal_Pole_Sup_L | -3.542 | 4.29E-04 | Frontal_Sup_2_L | Frontal_Sup_2_R | -3.232 | 1.30E-03 |
| Temporal_Sup_L | Temporal_Pole_Sup_L | -3.499 | 5.02E-04 | Precentral_L | Frontal_Inf_Orb_2_L | -3.233 | 1.29E-03 |
| Parietal_Inf_L | Temporal_Inf_R | -3.523 | 4.59E-04 | OFCmed_R | Cingulate_Mid_R | -3.223 | 1.34E-03 |
| Rolandic_Oper_R | OFCpost_L | -3.466 | 5.66E-04 | Hippocampus_L | Fusiform_R | -3.223 | 1.34E-03 |
| Frontal_Inf_Orb_2_R | Cingulate_Mid_L | -3.467 | 5.65E-04 | Frontal_Inf_Orb_2_L | Postcentral_L | -3.223 | 1.34E-03 |
| Insula_L | Hippocampus_L | -3.475 | 5.48E-04 | Supp_Motor_Area_L | Pallidum_L | -3.222 | 1.34E-03 |
| Frontal_Inf_Orb_2_L | Cuneus_R | -3.480 | 5.39E-04 | Amygdala_R | Fusiform_R | -3.218 | 1.36E-03 |
| Frontal_Mid_2_L | Occipital_Inf_R | -3.475 | 5.48E-04 | Olfactory_R | Occipital_Sup_R | -3.214 | 1.38E-03 |
| Rectus_R | Postcentral_R | -3.465 | 5.68E-04 | Frontal_Inf_Orb_2_R | SupraMarginal_L | -3.214 | 1.38E-03 |
| Olfactory_R | Parietal_Sup_R | -3.483 | 5.31E-04 | Supp_Motor_Area_R | Pallidum_L | -3.215 | 1.38E-03 |
| Frontal_Med_Orb_L | Parietal_Sup_R | -3.485 | 5.27E-04 | Frontal_Inf_Tri_L | Calcarine_R | -3.209 | 1.40E-03 |
| Frontal_Sup_2_R | Parietal_Inf_L | -3.473 | 5.51E-04 | Precentral_R | Lingual_L | -3.209 | 1.40E-03 |
| Amygdala_R | SupraMarginal_L | -3.469 | 5.60E-04 | Frontal_Inf_Orb_2_R | Occipital_Inf_L | -3.208 | 1.41E-03 |
|  |  |  |  | Frontal_Inf_Oper_R | Angular_L | -3.206 | 1.42E-03 |
