# Supplementary material for: Decreased brain connectivity in smoking contrasts with increased connectivity in drinking
Source: eLife. 2019 Jan 8;8:e40765. doi: 10.7554/eLife.40765 (PMC6336408; doi:10.7554/eLife.40765)
Supplement: Figure 3—source data 1. [file elife-40765-fig3-data1.docx]

**Figure 3—figure source data 1.** All significant links (FDR p<0.05) between the AAL2 areas for the drinking group.

| **Functional connectivity** | | **t value** | **p value** | **Functional connectivity** | | **t value** | **p value** |
| --- | --- | --- | --- | --- | --- | --- | --- |
| Precentral_L | OFCmed_L | -3.445 | 6.02E-04 | Cingulate_Ant_R | Heschl_R | -3.044 | 2.42E-03 |
| Precentral_R | OFCmed_L | -3.689 | 2.41E-04 | Cingulate_Mid_L | Heschl_R | -3.182 | 1.52E-03 |
| Rolandic_Oper_R | OFCmed_L | -3.851 | 1.28E-04 | Cingulate_Mid_R | Heschl_R | -3.105 | 1.97E-03 |
| Olfactory_L | OFCmed_L | -4.112 | 4.35E-05 | Heschl_L | Heschl_R | -3.281 | 1.08E-03 |
| Rectus_R | OFCmed_L | -3.611 | 3.25E-04 | Frontal_Inf_Orb_2_L | Temporal_Sup_L | -3.215 | 1.36E-03 |
| Frontal_Mid_2_R | OFCmed_R | -3.442 | 6.10E-04 | Frontal_Inf_Orb_2_R | Temporal_Sup_L | -3.227 | 1.31E-03 |
| OFCmed_L | OFCpost_L | -3.540 | 4.25E-04 | Frontal_Sup_Medial_L | Temporal_Sup_L | -3.057 | 2.31E-03 |
| Precentral_L | Cingulate_Ant_L | -3.523 | 4.52E-04 | Frontal_Sup_Medial_R | Temporal_Sup_L | -3.118 | 1.89E-03 |
| Precentral_R | Cingulate_Ant_L | -3.680 | 2.50E-04 | OFCmed_R | Temporal_Sup_L | -3.163 | 1.62E-03 |
| Rolandic_Oper_L | Cingulate_Ant_L | -3.528 | 4.44E-04 | OFCpost_L | Temporal_Sup_L | -3.216 | 1.36E-03 |
| Rolandic_Oper_R | Cingulate_Ant_L | -3.819 | 1.45E-04 | OFCpost_R | Temporal_Sup_L | -3.124 | 1.85E-03 |
| Insula_R | Cingulate_Ant_L | -3.481 | 5.27E-04 | Insula_L | Temporal_Sup_L | -2.967 | 3.10E-03 |
| Rolandic_Oper_R | Cingulate_Ant_R | -3.484 | 5.22E-04 | Pallidum_L | Temporal_Sup_L | -3.060 | 2.29E-03 |
| OFCmed_L | Cingulate_Ant_R | -3.478 | 5.34E-04 | Frontal_Inf_Orb_2_R | Temporal_Sup_R | -3.003 | 2.76E-03 |
| Precentral_R | Cingulate_Mid_L | -3.458 | 5.75E-04 | OFCmed_L | Temporal_Sup_R | -3.009 | 2.71E-03 |
| Cingulate_Ant_L | Postcentral_L | -3.598 | 3.42E-04 | Frontal_Inf_Orb_2_R | Temporal_Pole_Sup_L | -3.097 | 2.03E-03 |
| Cingulate_Mid_L | Postcentral_L | -3.756 | 1.86E-04 | Frontal_Inf_Tri_R | Rolandic_Oper_R | -2.936 | 3.43E-03 |
| OFCmed_L | Postcentral_R | -3.511 | 4.73E-04 | Frontal_Inf_Oper_R | Olfactory_R | -2.947 | 3.31E-03 |
| Cingulate_Ant_L | Postcentral_R | -3.658 | 2.72E-04 | Insula_L | Hippocampus_L | -2.946 | 3.32E-03 |
| Cingulate_Mid_L | Postcentral_R | -3.745 | 1.94E-04 | OFCpost_L | Hippocampus_R | -2.951 | 3.27E-03 |
| Cingulate_Ant_L | SupraMarginal_R | -3.472 | 5.47E-04 | OFCpost_L | Fusiform_L | -2.950 | 3.27E-03 |
| OFCmed_L | Heschl_L | -3.834 | 1.36E-04 | Frontal_Inf_Orb_2_R | Fusiform_R | -2.940 | 3.38E-03 |
| Cingulate_Ant_L | Heschl_L | -3.803 | 1.55E-04 | OFCpost_L | Postcentral_L | -2.940 | 3.39E-03 |
| Cingulate_Mid_L | Heschl_L | -3.495 | 5.02E-04 | OFCpost_L | Heschl_L | -2.937 | 3.41E-03 |
| Cingulate_Mid_R | Heschl_L | -3.544 | 4.19E-04 | Supp_Motor_Area_R | Heschl_R | -2.937 | 3.42E-03 |
| SupraMarginal_R | Heschl_L | -3.661 | 2.68E-04 | Rolandic_Oper_R | OFCpost_R | -2.928 | 3.52E-03 |
| SupraMarginal_R | Heschl_R | -3.575 | 3.72E-04 | Frontal_Sup_Medial_L | Postcentral_L | -2.923 | 3.57E-03 |
| Cingulate_Ant_L | Temporal_Sup_L | -3.812 | 1.49E-04 | Precentral_L | Frontal_Inf_Orb_2_L | -2.886 | 4.01E-03 |
| Cingulate_Ant_R | Temporal_Sup_L | -3.464 | 5.61E-04 | Rolandic_Oper_R | Frontal_Sup_Medial_L | -2.898 | 3.86E-03 |
| Cingulate_Mid_R | Temporal_Sup_L | -3.521 | 4.55E-04 | Frontal_Mid_2_L | OFCmed_L | -2.900 | 3.84E-03 |
| OFCmed_L | Temporal_Sup_L | -3.394 | 7.26E-04 | Frontal_Mid_2_R | OFCmed_L | -2.899 | 3.85E-03 |
| Frontal_Inf_Oper_L | OFCmed_L | -3.341 | 8.77E-04 | OFCmed_R | Insula_R | -2.890 | 3.96E-03 |
| Frontal_Inf_Oper_R | OFCmed_L | -3.345 | 8.63E-04 | Frontal_Inf_Oper_L | Cingulate_Ant_R | -2.885 | 4.02E-03 |
| Supp_Motor_Area_L | OFCmed_L | -3.354 | 8.37E-04 | Frontal_Sup_2_R | ParaHippocampal_R | -2.891 | 3.95E-03 |
| Cingulate_Ant_L | SupraMarginal_L | -3.354 | 8.37E-04 | OFCmed_R | Parietal_Sup_L | -2.909 | 3.73E-03 |
| Cingulate_Ant_R | Heschl_L | -3.365 | 8.04E-04 | Cingulate_Mid_R | SupraMarginal_L | -2.888 | 3.99E-03 |
| Frontal_Inf_Orb_2_R | Rolandic_Oper_R | -3.302 | 1.00E-03 | Rolandic_Oper_L | SupraMarginal_R | -2.884 | 4.04E-03 |
| Olfactory_R | Insula_R | -3.305 | 9.93E-04 | OFCpost_L | SupraMarginal_R | -2.887 | 3.99E-03 |
| Cingulate_Mid_L | Temporal_Sup_L | -3.302 | 1.00E-03 | Postcentral_R | Pallidum_L | -2.887 | 4.00E-03 |
| Frontal_Inf_Orb_2_L | Rolandic_Oper_L | -3.085 | 2.11E-03 | Rolandic_Oper_R | Pallidum_R | -2.904 | 3.80E-03 |
| Frontal_Inf_Orb_2_L | Rolandic_Oper_R | -3.045 | 2.41E-03 | Cingulate_Ant_L | Pallidum_R | -2.893 | 3.93E-03 |
| Precentral_L | Frontal_Sup_Medial_L | -3.015 | 2.66E-03 | OFCpost_R | Heschl_L | -2.900 | 3.84E-03 |
| Rolandic_Oper_R | Frontal_Sup_Medial_R | -3.126 | 1.84E-03 | Pallidum_R | Heschl_R | -2.897 | 3.88E-03 |
| Rolandic_Oper_L | OFCmed_L | -2.978 | 3.00E-03 | Heschl_L | Temporal_Sup_L | -2.901 | 3.83E-03 |
| Supp_Motor_Area_R | OFCmed_L | -3.063 | 2.27E-03 | Rolandic_Oper_L | OFCpost_L | -2.876 | 4.14E-03 |
| Frontal_Sup_Medial_R | OFCmed_L | -3.040 | 2.45E-03 | OFCmed_L | Calcarine_R | -2.865 | 4.29E-03 |
| Rolandic_Oper_R | OFCmed_R | -3.021 | 2.60E-03 | Frontal_Inf_Oper_L | Frontal_Inf_Tri_L | -2.862 | 4.33E-03 |
| Precentral_L | OFCpost_L | -3.079 | 2.15E-03 | Insula_L | Cingulate_Ant_R | -2.852 | 4.46E-03 |
| Rolandic_Oper_R | OFCpost_L | -3.025 | 2.57E-03 | Precentral_R | Pallidum_L | -2.849 | 4.50E-03 |
| Rolandic_Oper_L | Insula_L | -3.110 | 1.94E-03 | Rolandic_Oper_R | Pallidum_L | -2.850 | 4.49E-03 |
| OFCmed_L | Insula_L | -3.034 | 2.49E-03 | Precentral_L | Frontal_Sup_Medial_R | -2.847 | 4.54E-03 |
| OFCmed_L | Insula_R | -3.056 | 2.32E-03 | Frontal_Sup_Medial_L | OFCmed_L | -2.827 | 4.82E-03 |
| Frontal_Inf_Oper_L | Cingulate_Ant_L | -2.977 | 3.00E-03 | OFCmed_L | Fusiform_L | -2.839 | 4.65E-03 |
| Supp_Motor_Area_R | Cingulate_Ant_L | -3.143 | 1.74E-03 | OFCmed_L | Fusiform_R | -2.838 | 4.66E-03 |
| OFCmed_L | Cingulate_Ant_L | -3.077 | 2.16E-03 | OFCpost_L | Postcentral_R | -2.835 | 4.71E-03 |
| Insula_L | Cingulate_Ant_L | -3.133 | 1.80E-03 | Olfactory_R | Caudate_R | -2.832 | 4.74E-03 |
| Precentral_L | Cingulate_Ant_R | -3.193 | 1.47E-03 | ParaHippocampal_R | Caudate_R | -2.829 | 4.79E-03 |
| Precentral_R | Cingulate_Ant_R | -3.074 | 2.19E-03 | Frontal_Sup_Medial_R | Heschl_L | -2.828 | 4.80E-03 |
| Rolandic_Oper_L | Cingulate_Ant_R | -3.175 | 1.56E-03 | Frontal_Inf_Orb_2_R | Temporal_Pole_Sup_R | -2.831 | 4.76E-03 |
| Insula_R | Cingulate_Ant_R | -3.091 | 2.07E-03 | OFClat_R | Angular_R | 2.825 | 4.85E-03 |
| Precentral_L | Cingulate_Mid_L | -3.139 | 1.76E-03 | Frontal_Inf_Oper_L | OFCpost_L | -2.805 | 5.15E-03 |
| Rolandic_Oper_L | Cingulate_Mid_L | -3.169 | 1.59E-03 | OFCmed_L | Cingulate_Mid_L | -2.803 | 5.19E-03 |
| Rolandic_Oper_R | Cingulate_Mid_L | -3.067 | 2.24E-03 | OFCmed_L | Lingual_L | -2.810 | 5.08E-03 |
| Precentral_R | Cingulate_Mid_R | -3.256 | 1.18E-03 | Cingulate_Ant_L | Parietal_Sup_R | -2.807 | 5.12E-03 |
| Rolandic_Oper_L | Cingulate_Mid_R | -3.239 | 1.25E-03 | OFCmed_L | SupraMarginal_R | -2.814 | 5.02E-03 |
| Rolandic_Oper_R | Cingulate_Mid_R | -3.073 | 2.20E-03 | ParaHippocampal_R | Caudate_L | -2.805 | 5.16E-03 |
| Frontal_Inf_Orb_2_R | Hippocampus_R | -3.046 | 2.40E-03 | Hippocampus_L | Pallidum_R | -2.808 | 5.11E-03 |
| Frontal_Sup_Medial_R | Hippocampus_R | -3.175 | 1.56E-03 | Insula_L | Heschl_L | -2.813 | 5.03E-03 |
| OFCmed_L | ParaHippocampal_L | -2.995 | 2.83E-03 | Frontal_Inf_Oper_R | Frontal_Inf_Tri_R | -2.785 | 5.49E-03 |
| OFCpost_L | ParaHippocampal_L | -3.009 | 2.71E-03 | Precentral_L | Frontal_Inf_Orb_2_R | -2.794 | 5.34E-03 |
| Frontal_Inf_Orb_2_R | ParaHippocampal_R | -3.052 | 2.35E-03 | Frontal_Inf_Oper_L | Frontal_Sup_Medial_L | -2.777 | 5.62E-03 |
| Frontal_Sup_Medial_R | ParaHippocampal_R | -3.169 | 1.59E-03 | Precentral_L | OFCmed_R | -2.776 | 5.63E-03 |
| OFCpost_R | ParaHippocampal_R | -3.098 | 2.02E-03 | Frontal_Inf_Oper_R | Cingulate_Ant_L | -2.784 | 5.51E-03 |
| OFCmed_L | Calcarine_L | -2.977 | 3.00E-03 | Cingulate_Mid_L | Hippocampus_L | -2.785 | 5.49E-03 |
| OFCmed_L | Lingual_R | -2.989 | 2.89E-03 | Frontal_Inf_Orb_2_R | Amygdala_R | -2.776 | 5.63E-03 |
| Frontal_Inf_Orb_2_R | Fusiform_L | -3.071 | 2.21E-03 | Supp_Motor_Area_R | Pallidum_L | -2.775 | 5.65E-03 |
| Frontal_Sup_Medial_R | Fusiform_L | -2.971 | 3.06E-03 | Occipital_Sup_R | Pallidum_L | -2.788 | 5.43E-03 |
| Frontal_Inf_Orb_2_L | Postcentral_L | -2.965 | 3.12E-03 | SupraMarginal_L | Pallidum_L | -2.796 | 5.30E-03 |
| OFCmed_L | Postcentral_L | -3.132 | 1.80E-03 | OFCmed_L | Thalamus_L | -2.783 | 5.52E-03 |
| Cingulate_Ant_R | Postcentral_L | -3.095 | 2.04E-03 | OFCmed_R | Heschl_L | -2.787 | 5.45E-03 |
| Cingulate_Mid_R | Postcentral_L | -3.262 | 1.15E-03 | Frontal_Inf_Orb_2_R | Heschl_R | -2.797 | 5.29E-03 |
| Frontal_Sup_Medial_L | Postcentral_R | -2.968 | 3.09E-03 | Frontal_Sup_Medial_R | Heschl_R | -2.779 | 5.58E-03 |
| Frontal_Sup_Medial_R | Postcentral_R | -2.963 | 3.14E-03 | Frontal_Inf_Orb_2_L | Temporal_Pole_Sup_L | -2.784 | 5.50E-03 |
| Cingulate_Ant_R | Postcentral_R | -2.987 | 2.91E-03 | Frontal_Sup_2_R | OFCmed_L | -2.770 | 5.75E-03 |
| Cingulate_Mid_R | Postcentral_R | -3.084 | 2.11E-03 | Rectus_L | OFCmed_L | -2.768 | 5.77E-03 |
| Cingulate_Post_L | Postcentral_R | -2.974 | 3.03E-03 | Precentral_L | Cingulate_Mid_R | -2.764 | 5.86E-03 |
| Cingulate_Ant_R | SupraMarginal_L | -3.152 | 1.68E-03 | Frontal_Mid_2_R | ParaHippocampal_R | -2.768 | 5.78E-03 |
| Rolandic_Oper_R | SupraMarginal_R | -3.123 | 1.86E-03 | Cingulate_Ant_R | ParaHippocampal_R | -2.761 | 5.90E-03 |
| Olfactory_R | SupraMarginal_R | -3.193 | 1.47E-03 | Cingulate_Post_R | Postcentral_R | -2.761 | 5.89E-03 |
| Hippocampus_L | SupraMarginal_R | -3.173 | 1.57E-03 | OFCmed_L | Parietal_Sup_L | -2.768 | 5.78E-03 |
| SupraMarginal_L | SupraMarginal_R | -3.122 | 1.86E-03 | Frontal_Inf_Orb_2_R | Heschl_L | -2.760 | 5.92E-03 |
| OFCmed_L | Precuneus_L | -2.997 | 2.81E-03 | Frontal_Sup_Medial_L | Heschl_L | -2.765 | 5.83E-03 |
| Cingulate_Mid_L | Paracentral_Lobule_L | -3.002 | 2.77E-03 | Rolandic_Oper_L | Frontal_Sup_Medial_L | -2.756 | 6.00E-03 |
| OFCmed_L | Caudate_L | -2.959 | 3.18E-03 | Frontal_Sup_2_R | OFCmed_R | -2.752 | 6.07E-03 |
| Cingulate_Mid_L | Pallidum_L | -3.013 | 2.67E-03 | Cingulate_Mid_R | SupraMarginal_R | -2.750 | 6.10E-03 |
| Hippocampus_L | Pallidum_L | -3.071 | 2.21E-03 | Cuneus_L | Pallidum_L | -2.752 | 6.07E-03 |
| ParaHippocampal_R | Pallidum_R | -3.116 | 1.90E-03 | Frontal_Inf_Orb_2_R | Rolandic_Oper_L | -2.744 | 6.20E-03 |
| SupraMarginal_L | Pallidum_R | -3.029 | 2.53E-03 | Frontal_Med_Orb_R | OFCmed_L | -2.736 | 6.36E-03 |
| SupraMarginal_R | Pallidum_R | -3.162 | 1.63E-03 | Cingulate_Ant_L | ParaHippocampal_R | -2.737 | 6.34E-03 |
| Putamen_R | Pallidum_R | -3.221 | 1.33E-03 | Frontal_Sup_Medial_R | Fusiform_R | -2.740 | 6.29E-03 |
| Pallidum_R | Thalamus_L | -2.979 | 2.99E-03 | Cingulate_Mid_L | SupraMarginal_R | -2.735 | 6.38E-03 |
| Pallidum_L | Heschl_L | -3.006 | 2.74E-03 | Cingulate_Ant_L | Paracentral_Lobule_L | -2.741 | 6.26E-03 |
| Pallidum_R | Heschl_L | -2.992 | 2.86E-03 | ParaHippocampal_L | Caudate_L | -2.737 | 6.34E-03 |
| Thalamus_R | Heschl_L | -2.994 | 2.84E-03 | Frontal_Inf_Orb_2_L | Heschl_L | -2.748 | 6.15E-03 |
| OFCmed_L | Heschl_R | -3.111 | 1.93E-03 | Frontal_Inf_Tri_R | Temporal_Sup_L | -2.740 | 6.28E-03 |
| OFCmed_R | Heschl_R | -2.960 | 3.17E-03 | Supp_Motor_Area_L | Cingulate_Ant_L | -2.733 | 6.42E-03 |
| Cingulate_Ant_L | Heschl_R | -3.107 | 1.96E-03 | Cingulate_Ant_L | Occipital_Sup_R | -2.728 | 6.51E-03 |
