# Supplementary material for: Decreased brain connectivity in smoking contrasts with increased connectivity in drinking
Source: eLife. 2019 Jan 8;8:e40765. doi: 10.7554/eLife.40765 (PMC6336408; doi:10.7554/eLife.40765)
Supplement: Figure 4—source data 2. [file elife-40765-fig4-data2.docx]

**Figure 4—figure source data 2.** For the FC links identified as involved in drinking (Table 2): comparison of functional connectivity between three groups: smoking only; drinking only; and both smoking and drinking in which a common baseline was used (a group with no smoking and low drinking). The details of these groups are in the text of main paper. Here, we only show the top 30 significant links identified in the HCP dataset as shown in Table 2. The last two columns show the difference for these FC for the contrast smoking only – drinking only.

| **FC (identified in the analysis of drinking shown in Table 2)** | **Drinking only group – Baseline group** | | **Both smoking and drinking group – Baseline group** | | **Smoking only – drinking only** | |
| --- | --- | --- | --- | --- | --- | --- |
|  | **t value** | **p value** | **t value** | **p value** | **t value** | **p value** |
| Precentral_L-- OFCmed_L | 2.047 | 4.13E-02 | -0.026 | 9.79E-01 | -2.154 | 3.22E-02 |
| Precentral_R-- OFCmed_L | 1.851 | 6.49E-02 | -0.386 | 7.00E-01 | -3.125 | 1.98E-03 |
| Rolandic_Oper_R-- OFCmed_L | 2.385 | 1.75E-02 | -0.107 | 9.15E-01 | -3.301 | 1.10E-03 |
| Olfactory_L-- OFCmed_L | 2.320 | 2.09E-02 | 0.075 | 9.41E-01 | -2.252 | 2.52E-02 |
| Rectus_R-- OFCmed_L | 1.320 | 1.87E-01 | 0.895 | 3.72E-01 | -1.980 | 4.87E-02 |
| Frontal_Mid_2_R-- OFCmed_R | 2.644 | 8.53E-03 | 0.705 | 4.82E-01 | -2.055 | 4.08E-02 |
| OFCmed_L-- OFCpost_L | 1.541 | 1.24E-01 | -0.353 | 7.25E-01 | -2.931 | 3.68E-03 |
| Precentral_L-- Cingulate_Ant_L | 2.334 | 2.01E-02 | -0.741 | 4.59E-01 | -2.594 | 1.00E-02 |
| Precentral_R-- Cingulate_Ant_L | 2.178 | 3.00E-02 | 0.287 | 7.74E-01 | -2.267 | 2.42E-02 |
| Rolandic_Oper_L-- Cingulate_Ant_L | 2.327 | 2.05E-02 | -1.180 | 2.39E-01 | -2.933 | 3.65E-03 |
| Rolandic_Oper_R-- Cingulate_Ant_L | 2.346 | 1.95E-02 | -0.776 | 4.39E-01 | -2.800 | 5.49E-03 |
| Insula_R-- Cingulate_Ant_L | 2.071 | 3.90E-02 | -0.942 | 3.47E-01 | -2.429 | 1.58E-02 |
| Rolandic_Oper_R-- Cingulate_Ant_R | 1.980 | 4.84E-02 | -0.790 | 4.30E-01 | -2.599 | 9.87E-03 |
| OFCmed_L-- Cingulate_Ant_R | 2.250 | 2.50E-02 | -0.803 | 4.23E-01 | -2.259 | 2.47E-02 |
| Precentral_R-- Cingulate_Mid_L | 2.932 | 3.56E-03 | 0.435 | 6.64E-01 | -2.887 | 4.21E-03 |
| Cingulate_Ant_L-- Postcentral_L | 2.031 | 4.29E-02 | 0.169 | 8.66E-01 | -1.886 | 6.04E-02 |
| Cingulate_Mid_L-- Postcentral_L | 3.051 | 2.44E-03 | 0.887 | 3.76E-01 | -2.806 | 5.39E-03 |
| OFCmed_L-- Postcentral_R | 1.721 | 8.61E-02 | -0.362 | 7.18E-01 | -2.880 | 4.31E-03 |
| Cingulate_Ant_L-- Postcentral_R | 2.232 | 2.61E-02 | 0.485 | 6.28E-01 | -1.676 | 9.49E-02 |
| Cingulate_Mid_L-- Postcentral_R | 3.339 | 9.21E-04 | 0.904 | 3.67E-01 | -2.216 | 2.76E-02 |
| Cingulate_Ant_L-- SupraMarginal_R | 2.106 | 3.58E-02 | -1.328 | 1.85E-01 | -2.660 | 8.31E-03 |
| OFCmed_L-- Heschl_L | 2.828 | 4.92E-03 | 0.009 | 9.93E-01 | -3.256 | 1.28E-03 |
| Cingulate_Ant_L-- Heschl_L | 2.702 | 7.19E-03 | -0.637 | 5.24E-01 | -2.916 | 3.85E-03 |
| Cingulate_Mid_L-- Heschl_L | 3.001 | 2.86E-03 | -0.175 | 8.61E-01 | -3.880 | 1.32E-04 |
| Cingulate_Mid_R-- Heschl_L | 2.775 | 5.78E-03 | 0.150 | 8.81E-01 | -3.217 | 1.46E-03 |
| SupraMarginal_R-- Heschl_L | 2.909 | 3.83E-03 | -0.231 | 8.18E-01 | -2.922 | 3.79E-03 |
| SupraMarginal_R-- Heschl_R | 2.852 | 4.57E-03 | -0.198 | 8.43E-01 | -2.383 | 1.79E-02 |
| Cingulate_Ant_L-- Temporal_Sup_L | 2.661 | 8.11E-03 | -0.497 | 6.20E-01 | -2.688 | 7.65E-03 |
| Cingulate_Ant_R-- Temporal_Sup_L | 2.347 | 1.94E-02 | -0.392 | 6.95E-01 | -2.719 | 6.98E-03 |
| Cingulate_Mid_R-- Temporal_Sup_L | 3.299 | 1.06E-03 | 0.547 | 5.85E-01 | -2.287 | 2.30E-02 |
