# Supplementary material for: Decreased brain connectivity in smoking contrasts with increased connectivity in drinking
Source: eLife. 2019 Jan 8;8:e40765. doi: 10.7554/eLife.40765 (PMC6336408; doi:10.7554/eLife.40765)
Supplement: Supplementary file 2. [file elife-40765-supp2.docx]

The demographic characteristics of participants from the IMAGEN dataset.

| **IMAGEN 19** | | | | | | |
| --- | --- | --- | --- | --- | --- | --- |
|  | **Non-smoker** | **Regular Smoker** | **Statistic* / p value** | **Low drinker** | **High Drinker** | **Statistic* / p value** |
| **Number of participants** | 295 | 180 | / | 378 | 566 | / |
| **Age** | 19 | 19 | / | 19 | 19 | / |
| **Gender (male/female)** | 128 / 167 | 100 / 80 | 6.63 / 0.010 | 157 / 221 | 281 / 285 | 6.00 / 0.0143 |
| **BMI** | 22.47±3.73 | 23.15±4.11 | -1.85 / 0.064 | 22.20±3.65 | 23.03±3.94 | -3.28 / 0.0011 |
| **Mean FD** | 0.177±0.053 | 0.187±0.054 | -2.04 / 0.042 | 0.174±0.054 | 0.185±0.054 | -3.06 / 0.0023 |
| **Smoking** **frequency** | / | 3.92±0.90 | / | 0.675±1.393 | 1.292±1.686 | -5.90 / <0.0001 |
| **Smoking amount** | / | 6 | / | 1.855±2.386 | 3.444±2.405 | -9.98 / <0.0001 |
| **Drinking frequency** | 3.078±1.836 | 4.783±1.590 | -10.32 / <0.0001 | 3.069±1.888 | 4.709±1.296 | -15.82 / <0.0001 |
| **Drinking amount** | 1.444±0.875 | 2.450±1.300 | -10.09 / <0.0001 | 0.939±0.239 | 2.751±0.929 | -37.09 / <0.0001 |
| **Impulsivity** | 10.41±1.96 | 11.84±2.19 | -7.36 / <0.0001 | 10.78±2.10 | 11.16±2.08 | -2.69 / 0.0073 |

| **IMAGEN 14** | | | | | | |
| --- | --- | --- | --- | --- | --- | --- |
|  | **Non-smoker** | **Regular Smoker** | **Statistic* / p value** | **Low drinker** | **High Drinker** | **Statistic* / p value** |
| **Number of participants** | 56 | 19 | / | 86 | 91 | / |
| **Age** | 14 | 14 | / | 14 | 14 | / |
| **Gender (male/female)** | 28 / 28 | 12 / 7 | 0.987 / 0.321 | 32 / 54 | 54 / 37 | 8.67 / 0.0032 |
| **BMI** | 21.95±3.75 | 22.46±3.42 | -0.52 / 0.603 | 21.67±3.17 | 22.77±4.20 | -1.95 / 0.052 |
| **Mean FD** | 0.176±0.059 | 0.185±0.065 | -0.54 / 0.591 | 0.168±0.061 | 0.171±0.063 | -0.32 / 0.732 |
| **Smoking frequency** **at 14** | 0 | 0 | / | 0.023±0.152 | 0.033±0.180 | -0.388 / 0.700 |
| **Smoking amount at 14** | 0 | 0 | / | 0.267±0.693 | 0.659±1.384 | -2.36 / 0.019 |
| **Smoking frequency at 19** | 0 | 3.947±0.970 | / | 0.849±1.538 | 1.297±1.560 | -1.92 / 0.056 |
| **Smoking amount at 19** | 0 | 6 | / | 2.035±2.466 | 3.462±2.433 | -3.87 / 0.0002 |
| **Drinking frequency at 14** | 0.750±1.014 | 0.947±0.911 | -0.751 / 0.455 | 0.907±0.903 | 1.374±1.262 | -2.82 / 0.0054 |
| **Drinking amount at 14** | 0.554±0.537 | 0.790±0.419 | -1.742 / 0.086 | 0.686±0.467 | 0.791±0.409 | -1.597 / 0.112 |
| **Drinking frequency at 19** | 2.607±1.816 | 4.579±1.539 | -4.24 / 0.0001 | 2.93±1.69 | 4.60±1.41 | -7.16 / <0.0001 |
| **Drinking amount at 19** | 1.304±0.807 | 1.737±1.046 | -1.87 / 0.065 | 0.930±0.256 | 2.593±0.816 | -18.07 / <0.0001 |
| **Impulsivity at 14** | 11.33±2.12 | 12.68±2.33 | -2.35 / 0.022 | 11.55±2.03 | 12.46±2.14 | -2.89 / 0.0043 |
| **Impulsivity at 19** | 10.29±2.17 | 11.26±1.94 | -1.74 / 0.086 | 10.48±2.13 | 11.44±2.43 | -2.80 / 0.0057 |

Values are n or mean ± SD.

*: A group difference (independent samples t test or χ^2^ test).
